# Breast cancer prediction with transcriptome profiling using feature selection and machine learning methods

**DOI:** 10.1186/s12859-022-04965-8

**Published:** 2022-10-01

**Authors:** Eskandar Taghizadeh, Sahel Heydarheydari, Alihossein Saberi, Shabnam JafarpoorNesheli, Seyed Masoud Rezaeijo

**Affiliations:** 1 Department of Medical Genetic, Faculty of Medicine, Ahvaz Jundishapur University of Medical Sciences, Ahvaz, Iran; 2 Department of Radiology Technology, Shoushtar Faculty of Medical Sciences, Shoushtar, Iran; 3 Faculty of Engineering, University of Science and Culture, Tehran, Iran; 4 Department of Medical Physics, Faculty of Medicine, Ahvaz Jundishapur University of Medical Sciences, Ahvaz, Iran

**Keywords:** Breast cancer, Prediction, Transcriptome profiling, Feature selection, Machine learning

## Abstract

**Background:**

We used a hybrid machine learning systems (HMLS) strategy that includes the extensive search for the discovery of the most optimal HMLSs, including feature selection algorithms, a feature extraction algorithm, and classifiers for diagnosing breast cancer. Hence, this study aims to obtain a high-importance transcriptome profile linked with classification procedures that can facilitate the early detection of breast cancer.

**Methods:**

In the present study, 762 breast cancer patients and 138 solid tissue normal subjects were included. Three groups of machine learning (ML) algorithms were employed: (i) four feature selection procedures are employed and compared to select the most valuable feature: (1) ANOVA; (2) Mutual Information; (3) Extra Trees Classifier; and (4) Logistic Regression (LGR), (ii) a feature extraction algorithm (Principal Component Analysis), iii) we utilized 13 classification algorithms accompanied with automated ML hyperparameter tuning, including (1) LGR; (2) Support Vector Machine; (3) Bagging; (4) Gaussian Naive Bayes; (5) Decision Tree; (6) Gradient Boosting Decision Tree; (7) K Nearest Neighborhood; (8) Bernoulli Naive Bayes; (9) Random Forest; (10) AdaBoost, (11) ExtraTrees; (12) Linear Discriminant Analysis; and (13) Multilayer Perceptron (MLP). For evaluating the proposed models' performance, balance accuracy and area under the curve (AUC) were used.

**Results:**

Feature selection procedure LGR + MLP classifier achieved the highest prediction accuracy and AUC (balanced accuracy: 0.86, AUC = 0.94), followed by an LGR + LGR classifier (balanced accuracy: 0.84, AUC = 0.94). The results showed that achieved AUC for the LGR + LGR classifier belonged to the 20 biomarkers as follows: TMEM212, SNORD115-13, ATP1A4, FRG2, CFHR4, ZCCHC13, FLJ46361, LY6G6E, ZNF323, KRT28, KRT25, LPPR5, C10orf99, PRKACG, SULT2A1, GRIN2C, EN2, GBA2, CUX2, and SNORA66.

**Conclusions:**

The best performance was achieved using the LGR feature selection procedure and MLP classifier. Results show that the 20 biomarkers had the highest score or ranking in breast cancer detection.

## Introduction

Worldwide, breast cancer is the most common cancer in women, including almost one-third of all females' malignancies. The risk of developing breast cancer is a multi-step process involving multiple cell types, and its prevention remains challenging worldwide [1]. Many risk factors such as aging, estrogen, family history, and gene mutations can increase the feasibility of developing breast cancer [2]. Detection of breast cancer may be hard at the beginning of the disease due to the absence of symptoms; after some clinical tests, an accurate diagnosis should be able to differentiate the benign and malignant tumors. Hence, an early breast cancer diagnosis is one of the best strategies to prevent this disease. In some developed countries, breast cancer patients have a 5 year relative survival rate above 80% due to early prevention [3, 4]. Although some improvements have been achieved in the treatment methods in recent years, late diagnosis and treatment resistance are serious problems that lead to poor prognoses for some patients [5–7].

Different factors are involved in promoting breast cancer; most implied changes in the expression of certain genes, such as microRNAs. MicroRNAs have the ability to control signaling pathways, hence affecting tumorigenesis and various aspects of cancer progression [8]. Evaluation of the expression profiles of genes and microRNAs can be applied as valuable clues in discovering new biomarkers which are more effective for early diagnosis of breast cancer and therapeutic strategies [9]. Machine Learning (ML) procedures can be considered as another strategy for discovering valuable information in large data [5]. ML is a type of artificial intelligence (AI) that develops to simulate human intelligence by learning from data and ongoing experience. This approach needs the integration of numerous data sets of biological information, allowing the design of a statistical model that estimates the unknown parameters [10]. Recently, ML has achieved considerable success in medicine, where it has been effective in the multi-pathology classification task. In breast cancer cases, applying ML concentrates on finding specific changes in gene expression that allows earlier diagnosis of the disease [11]. Thus, an extensive data set of microRNAs can be employed in breast cancer patients as potential biomarkers and utilized them in ML algorithms. Thereby, a model can be created to detect each illness. It is possible to predict the pathology more accurately and differentiate between patients with- and without breast cancer based on their information. In the present study, we used a hybrid machine learning systems (HMLS) strategy that includes the extensive search for the discovery of the most optimal HMLSs including feature selection algorithms, a feature extraction algorithm, and classifiers for diagnosing breast cancer. Hence, several experiments were conducted to select the best biomarkers with the highest ranking. Therefore, this study aims to obtain a high-importance biomarker linked with classification procedures that can facilitate the early detection of breast cancer. To get the best combination of feature selection and classification procedures, extensive comparative analyses were performed using performance metrics such as balanced accuracy, and receiver operator characteristic curve (AUC) statistics.

## Methods

### Patients

Clinical data from breast cancer patients were downloaded from the TCGA database. Criteria for including patient data in the present study include histopathological diagnosis of breast cancer and solid tissue normal. Totally 762 breast cancer patients and 138 solid tissue normal subjects were included. To overcome the issue of imbalanced data distribution, we applied an approach explained in the “Experimental design and Statistical analysis” section.

### ML methods

ML methods are tools utilized to create and evaluate algorithms that facilitate prediction and classification. ML is based on four steps: data collection, picking a model, training the model, and testing the model. In this study, three groups of algorithms were employed: feature selection, feature extraction, and classification algorithms. These are elaborated on next.

### Feature selection algorithms

ML procedures have difficulty in dealing with a large number of input features. Hence, to support the process of applying ML effectively in real-world scenarios, data preprocessing is an essential task. Feature selection is one of the most frequent procedures in data preprocessing [12]. It has become a vital element of the ML process to obtain the relevant feature or feature subsets in the literature to achieve their classification objectives. However, besides the advantages of feature selection procedures to search for a subset of relevant features, they are used to avoid overfitting and provide faster and more cost-effective models. In the present study, four feature selection procedures, including filter and embedded approaches, are employed and compared to select the most valuable feature: (1) ANOVA; (2) Mutual Information (MI); (3) Extra Trees Classifier (ETC); and (4) Logistic Regression (LGR).

### Feature extraction algorithm

We employed feature extraction to convert high-dimensional data to fewer dimensions; thus, the risk of overfitting was reduced. Dimensionality reduction procedures use no label for feature extraction; hence, they only rely on patterns between input features. Consistent with previous studies [13, 14], Principal Component Analysis (PCA) was outperformed other feature extraction algorithms. PCA is a dimensionality reduction procedure that generates new specified features, but not a feature selection procedure. PCA transforms features, but feature selection procedures choose features without transforming them. Hence, as the feature extractor, PCA was implemented in this study.

### Classifier algorithms

We selected 13 classification algorithms: (1) LGR; (2) Support Vector Machine (SVM); (3) Bagging, (4) Gaussian Naive Bayes (GNB); (5) Decision Tree (DT); (6) Gradient Boosting Decision Tree (GBDT); (7) K Nearest Neighborhood (KNN); (8) Bernoulli Naive Bayes (BNB); (9) Random Forest (RF), (10) AdaBoost, (11) ExtraTrees, (12) Linear Discriminant Analysis (LDA), and (13) Multilayer Perceptron (MLP). Of note, all the feature selection, extraction, and classification procedures were implemented using the scikit-learn package in python (scikit-learn version 1.0.2, python version 3.8.3). The cross-combination approach was employed to compare the performance of feature selection, extraction, and classification procedures. Therefore, each feature selection and the extraction procedure were combined with all the nine classification procedures. Finally, we got 65 combinations of ML strategies.

### Experimental design and statistical analysis

In this study, the performance of the ML procedures was obtained with five-fold cross-validation. Five-fold cross-validation split the data into five parts and then alternately used four parts for training and the rest for testing. Therefore, the original sample is randomly partitioned into five equal size subsamples.

A problem with unbalanced classification for a model is that there are few samples of the minority class to learn the decision boundary effectively. In other words, a dataset is unbalanced if the classification classes are not approximately equally represented. In our study, the dataset was unbalanced; the breast cancer group was higher than the solid tissue group, which means the unbalanced distribution of the data might bias. Hence, we used Synthetic Minority Over-sampling Technique (SMOTE) [15] to solve this problem. SMOTE is an oversampling method that makes synthetic minority class samples. It potentially performs better than simple oversampling. This method is designed for selecting samples close to the feature space, drawing a line between the samples in the feature space, and drawing a new sample at a point along that line. As mentioned in [15], it shows that SMOTE can achieve better performance when combined with the under-sampling of the majority class. Therefore, we employed the first oversample of the minority class (solid tissue group) with SMOTE, then undersample the majority class (breast cancer group). The performance of ML algorithms is commonly evaluated using predictive accuracy and is calculated as follows:

1 $${\text{Accuracy}} = \frac{{{\text{TP}} + {\text{TN}}}}{{{\text{TP}} + {\text{FP}} + {\text{TN}} + {\text{FN}}}}$$

In the context of balanced datasets, it is reasonable to use accuracy as a performance metric. However, this is not appropriate when the dataset is unbalanced. Hence, for evaluating the proposed models' performance, balance accuracy defined in Eq. (4) and area under curve (AUC) were used as diagnostic indicators.

2 $${\text{Sensitivity}} = \frac{{{\text{TP}}}}{{{\text{TP}} + {\text{FN}}}}$$

3 $${\text{Specificity}} = \frac{{{\text{TN}}}}{{{\text{TN}} + {\text{FP}}}}$$

4 $${\text{Balanced}}\;{\text{accuracy}} = \frac{{{\text{Sensitivity}} + {\text{Specificity}}}}{2}$$

## Results

**Fig. 1:**
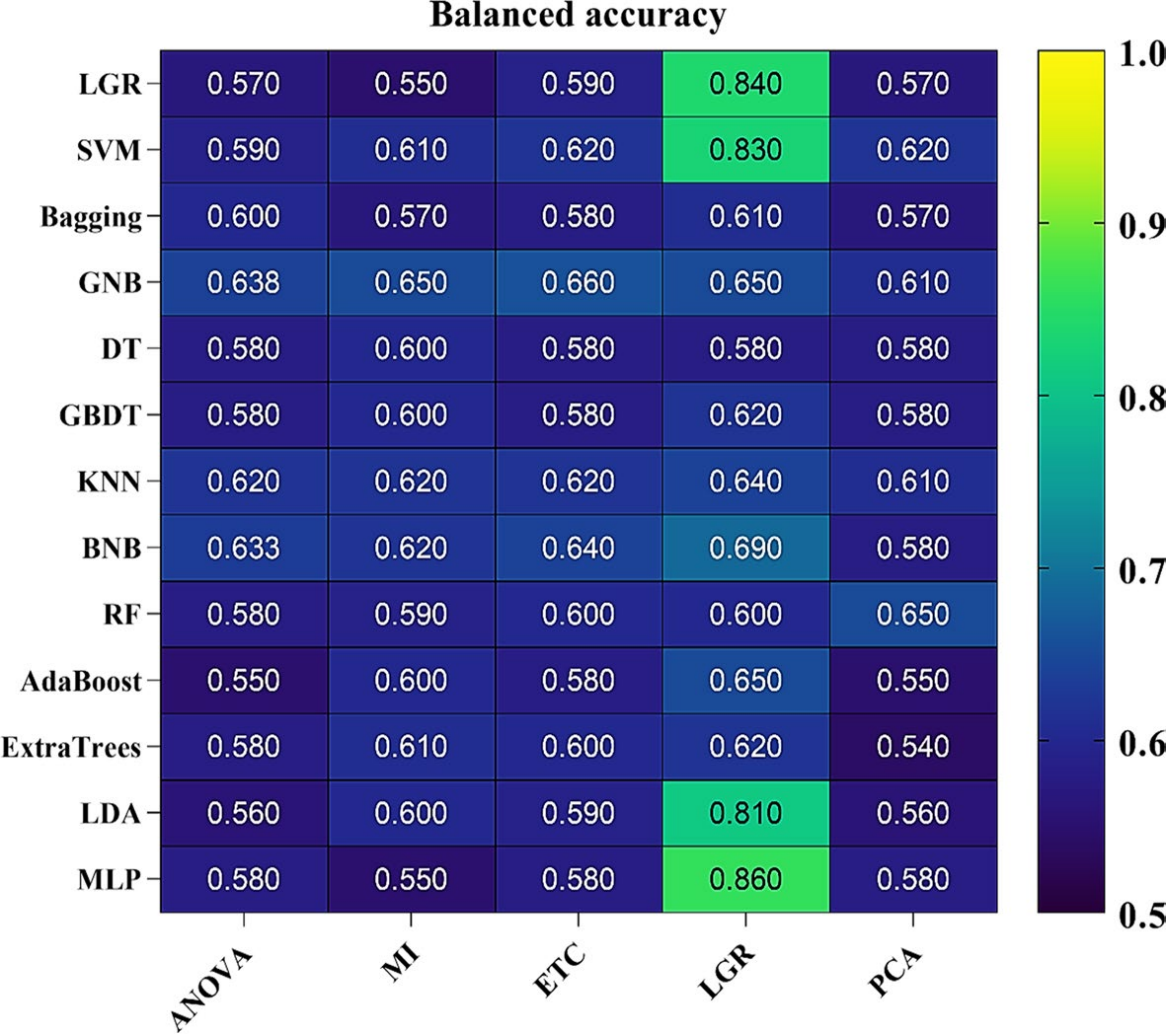
Balanced accuracy heatmap of the feature selection and classification procedures

**Fig. 2:**
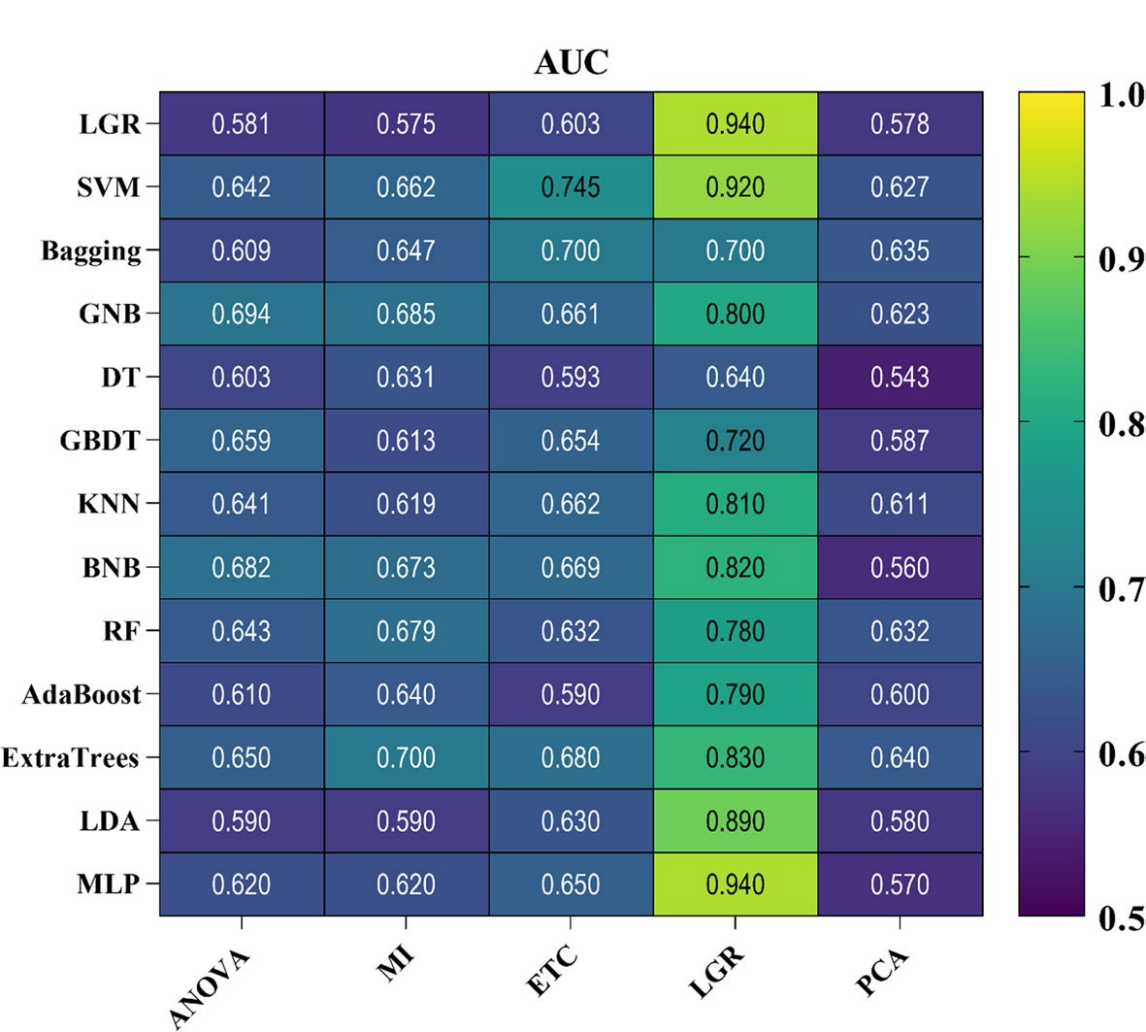
AUC heatmap of the feature selection and classification procedures

**Fig. 3:**
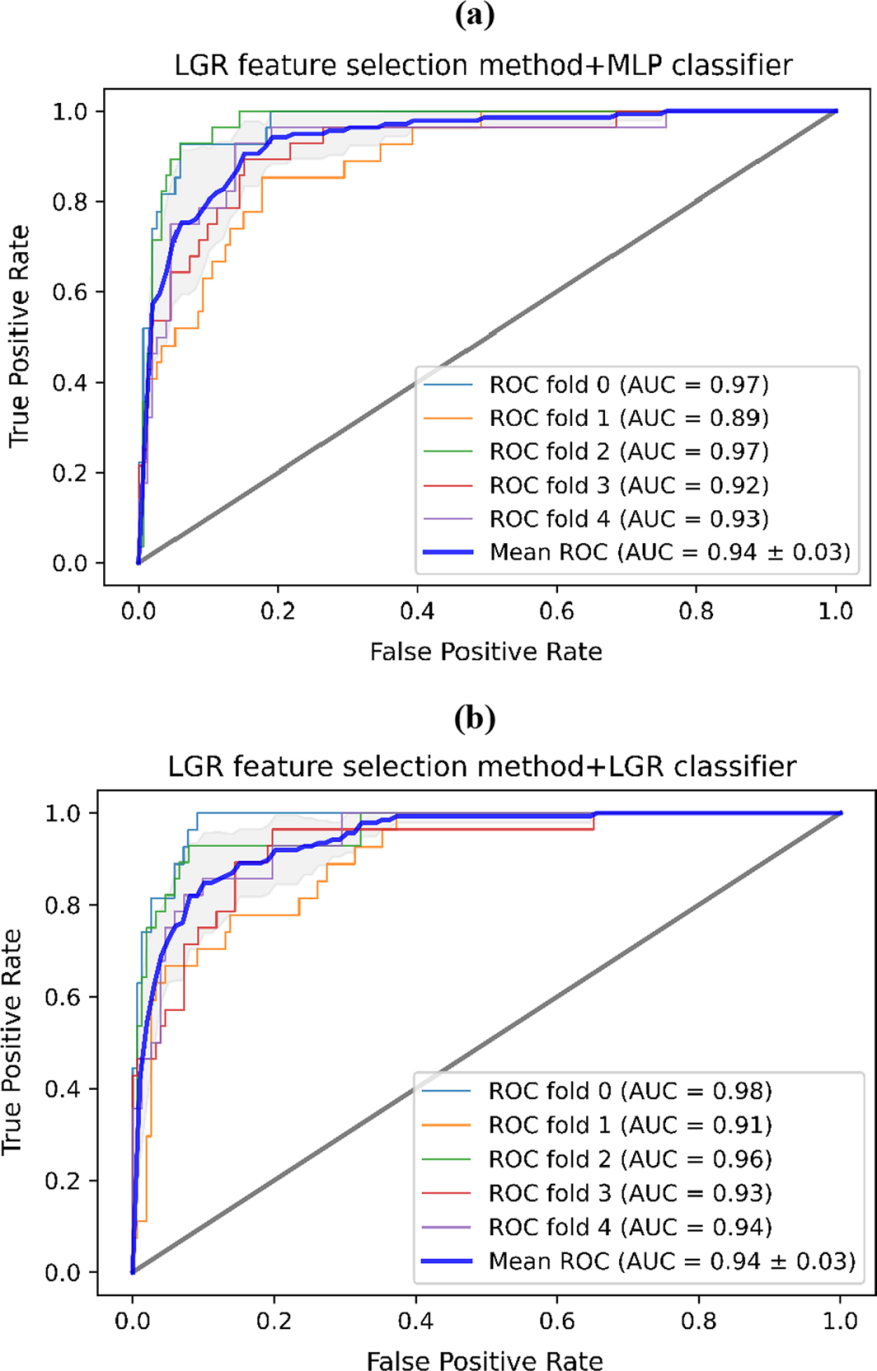
ROC curves for the max AUC of the classification procedures

The diagnostic performance of ML procedures was evaluated by five-fold cross-validation and we examined 65 combinations of ML procedures. Figures 1 and 2 show the balanced accuracy and AUC results as heatmaps in five-fold cross-validation. Feature selection procedure LGR + MLP classifier achieved the highest prediction accuracy and AUC (balanced accuracy: 0.86, AUC = 0.94), followed by an LGR + LGR classifier (balanced accuracy: 0.84, AUC = 0.94). The ROC plot for the mentioned models is shown in Fig. 3. The results showed that achieved AUC for the two classifiers belonged to the 20 biomarkers. In contrast, the LGR classifier had low AUC and balanced accuracy scores for the ANOVA and MI feature selections. More details about selected biomarkers are given in Table 1. It is necessary to mention that the LGR feature selection procedure presented max balanced accuracy and AUC performance for all classifiers. We observed that except for the DT classifier, classifiers had a mean AUC ≥ 0.7. Specifically, the AUC for LGR classifier was 0.94 (SD, 0.03), for SVM classifier was 0.92 (SD, 0.03), for Bagging classifier was 0.70 (SD, 0.05), for GNB classifier was 0.80 (SD, 0.04), for GBDT classifier was 0.72 (SD, 0.03), for KNN classifier was 0.81 (SD, 0.04), for BNB classifier was 0.82 (SD, 0.04), for RF classifier was 0.78 (SD, 0.03), for AdaBoost classifier was 0.79 (SD, 0.03), for ExtraTrees classifier was 0.83 (SD, 0.02), for LDA classifier was 0.89 (SD, 0.04), and for MLP classifier was 0.94 (SD, 0.04). In addition, MLP classifier [0.86 (SD, 0.04)], LDA classifier [0.81 (SD, 0.03)], BNB classifier [0.69 (SD, 0.04)], and GNB classifier [0.65 (SD, 0.04)] also had high balanced accuracy.

**Table 1:** Selected biomarkers using LGR feature selection method

No.	Type of biomarker	No.	Type of biomarker
1	TMEM212	11	KRT25
2	SNORD115-13	12	LPPR5
3	ATP1A4	13	C10orf99
4	FRG2	14	PRKACG
5	CFHR4	15	SULT2A1
6	ZCCHC13	16	GRIN2C
7	FLJ46361	17	EN2
8	LY6G6E	18	GBA2
9	ZNF323	19	CUX2
10	KRT28	20	SNORA66

This plot calculates the classification performance at various decision threshold values by showing the model’s ability at distinguishing both solid tissue group and breast cancer group. As the blue curve, the mean AUC of folds, gets closer to the upper and left-hand boundaries, the model indicates a smaller error. The black line represents the case in which the model is unable to tell one class from the other.

## Discussion

In global terms, breast cancer has the second annual incidence and the fifth mortality rate of prevalent cancers [16]. Prior studies indicate that genetic, epigenetic, and environmental factors could be correlated with the initiation and progression of breast cancer [5]. Based on the molecular characteristics, histopathological form, and clinical outcome, breast cancer is classified into different types. Recognizing genes associated with breast cancer development and prognosis and clarifying the underlying molecular mechanisms seems needed [17]. Hence, in breast cancer, bioinformatic analysis is utilized to identify potential pathogenic and differentially expressed genes [18]. Recently, there have been many studies of models for analyzing omics data. Especially the success of ML methods in processing a large amount of data has revolutionized bioinformatics and conventional forms of genetic diagnosis [19].

While the ML methods in computational biology are still in their infancy, a general approach for identifying molecular biomarkers is screening biomarkers by gene expression analysis. Liu et al. [10] aimed to propose a method to predict molecular subtypes using a hybrid neural network based on multimodal data. The proposed method integrated gene expression data, copy number variants, and pathological images to accurately predict molecular subtypes. The proposed architecture yields 88.07% of classification accuracy and an AUC of 94.2% for each subtype. In the present study, we used clinical data from breast cancer patients, and the maximum AUC of our results for the LGR and MLP classifiers agreed with the study by Liu et al. [10]. Recently, microRNAs have been utilized as biomarkers due to their beneficial role in breast cancer diagnosis. Hence, Yerukala et al. [20] propose an SVM classifier based on microRNAs to categorize patients with breast cancer into early and advanced stages. Their classifier uses a feature selection method to identify a small set of informative microRNAs. The method identified 34 of 503 microRNAs as a signature and achieved a mean accuracy of 80.38%. In this study, using the LGR feature selection, 20 biomarkers from 16,380 were selected to maximize prediction accuracy. Of note, in contrast with the study by Yerukala et al. [20], the maximum accuracy was achieved using the MLP classifier, an accuracy of 86%. It is worth emphasizing that our study evaluated different ML procedures. The expression of microRNA in breast cancer patients indicates a pattern compared to normal breasts, hence, showing its role as a potential diagnostic marker. However, not all microRNA profiles have a significant role in cancer detection. It is necessary to mention that selecting features by removing unnecessary features will improve accuracy and reduce the complexity of the machine learning models. Hence, Adorada et al. [21] used the SVM-RFE and univariate selection to select microRNA expression in breast cancer. They used 248 samples containing 1881 microRNA expressions as feature profiles. Feature selection was performed by selecting ten features that have the highest rating. In agreement with the study by Adorada et al. [21], we applied four feature selection procedures and a feature extraction algorithm to select the best feature. Although the sample size was relatively large in this study, training a multi-center study in the future can give an additional understanding of the reproducibility of machine learning methods. Therefore, future studies with a large sample size are suggested.

## Conclusions

We presented an HMLS strategy that includes an extensive search to discover the most optimal HMLS for diagnosing breast cancer. The best performance was achieved using the LGR feature selection procedure and MLP classifier (balanced accuracy: 0.86, AUC = 0.94). Results show the 20 biomarkers had the highest score or ranking in breast cancer detection.

## Data Availability

Data and code about the results of this study are publicly shared at: https://github.com/MASOUD-AJUMS/Breast-cancer-prediction-/tree/main.
